# Radial Basis Function for Breast Lesion Detection from MammoWave Clinical Data

**DOI:** 10.3390/diagnostics11101930

**Published:** 2021-10-18

**Authors:** Soumya Prakash Rana, Maitreyee Dey, Riccardo Loretoni, Michele Duranti, Lorenzo Sani, Alessandro Vispa, Mohammad Ghavami, Sandra Dudley, Gianluigi Tiberi

**Affiliations:** 1 School of Engineering, London South Bank University, London SE1 0AA, UK; deym5@lsbu.ac.uk (M.D.); ghavamim@lsbu.ac.uk (M.G.); dudleyms@lsbu.ac.uk (S.D.); tiberig@lsbu.ac.uk (G.T.); 2 Breast Screening and Diagnostic Breast Cancer Unit, AUSL Umbria 2, 06034 Foligno, Italy; riccardo.loretoni@uslumbria2.it; 3 Department of Diagnostic Imaging, Perugia Hospital, 06156 Perugia, Italy; michele.duranti@ospedale.perugia.it; 4 UBT-Umbria Bioengineering Technologies, 06081 Perugia, Italy; l.sani@ubt-tech.com (L.S.); a.vispa@ubt-tech.com (A.V.)

**Keywords:** MammoWave, breast lesion detection, machine learning

## Abstract

Recently, a novel microwave apparatus for breast lesion detection (MammoWave), uniquely able to function in air with 2 antennas rotating in the azimuth plane and operating within the band 1–9 GHz has been developed. Machine learning (ML) has been implemented to understand information from the frequency spectrum collected through MammoWave in response to the stimulus, segregating breasts with and without lesions. The study comprises 61 breasts (from 35 patients), each one with the correspondent output of the radiologist’s conclusion (i.e., gold standard) obtained from echography and/or mammography and/or MRI, plus pathology or 1-year clinical follow-up when required. The MammoWave examinations are performed, recording the frequency spectrum, where the magnitudes show substantial discrepancy and reveals dissimilar behaviours when reflected from tissues with/without lesions. Principal component analysis is implemented to extract the unique quantitative response from the frequency response for automated breast lesion identification, engaging the support vector machine (SVM) with a radial basis function kernel. In-vivo feasibility validation (now ended) of MammoWave was approved in 2015 by the Ethical Committee of Umbria, Italy (N. 6845/15/AV/DM of 14 October 2015, N. 10352/17/NCAV of 16 March 2017, N 13203/18/NCAV of 17 April 2018). Here, we used a set of 35 patients. According to the radiologists conclusions, 25 breasts without lesions and 36 breasts with lesions underwent a MammoWave examination. The proposed SVM model achieved the accuracy, sensitivity, and specificity of 91%, 84.40%, and 97.20%. The proposed ML augmented MammoWave can identify breast lesions with high accuracy.

## 1. Introduction

Mammography is considered as the gold standard technology for breast screening, where age and screening frequency are defined by contemplating the mammography risk-benefit ratio [1,2]. Indeed, risks associated with X-ray cumulative effects (and low sensitivity in dense breasts) limit the use of mammography—usually, women in the range between 50–69 years are invited for screening once every two or three years [3,4,5]. Usually, women after the age of 49 are offered bi/tri-annual screening to reduce the impact of ionizing radiation. Although, the recent studies reported that lowering the screening age limit to 40 years could potentially reduce breast cancer mortality rates [6,7,8].

Microwave-based techniques have recently been developed as a potential breast screening tool [9,10,11,12]. Microwave-based techniques are non-ionizing, non-invasive, and painless since they do not involve breast compression during screening. Microwave-based systems utilize the contrast in dielectric properties, i.e., permittivity and conductivity, within the spectrum of microwave frequencies (i.e., approximately in the range of 1 and 10 GHz) between healthy tissues and tissues with lesions. A high difference in one or both dielectric properties (up to 5) [13] stated between healthy tissues and tissues with lesions; newer studies confirm such high contrast exists between fatty breast tissues and lesions (WF), while it declines when considering fibro glandular breast tissues [14,15]. Wide-ranging research on microwave-based procedures began in the late 1990’s, with a number of different prototypes developed [16]. Hitherto, few clinically tested microwave breast imaging operational systems have been reported in the literature, which was developed by Dartmouth College, USA [17], the University of Bristol, UK, jointly with Micrima Limited, UK [18,19,20], UBT Srl, Italy [21], University of Calgary, CA, [22,23], Southern University of Science and Technology, China [24], Hiroshima University, Japan, [25], McGill University, Canada [26], and Shizuoka University, Japan [27]. Only two of the aforementioned systems methods have now cleared a regulatory path (CE marking), i.e., MARIA (Micrima Limited, UK) and MammoWave^®^ (UBT Srl, Perugia, Italy). One of these models, the MARIA system, utilizes an array of 60 antennas (operating within the 3–8 GHz frequency band) and a matching liquid to perform the radar approach with a sensitivity of 76% [18,19,20]. MammoWave is uniquely skilled to work in the air with two antennas rotating in the azimuth plane, operates within the frequency band of 1–9 GHz. MammoWave examinations are performed in a multi-bistatic fashion, measuring the complex $S_{21}$ in the frequency domain. In more detail, the device transmits non-invasive and low-power microwave signals through the breast and accumulates the backscattered signatures (commonly denoted as the $S_{21}$ signals in engineering terminology) from a plurality of angular directions. A sensitivity of up to 82% has been reported [28]. An initial Machine Learning (ML) experiment [21] was performed on a limited number of subjects employing popular ML tools to classify the frequency response signal backscatter from the breast with radiological findings (WF) and no radiological findings (NF); it was found that the support vector machine (SVM) with a quadratic kernel outperformed the various applied methods tested.

The aim of this paper is to apply principal component analyses (PCA) to extract the unique quantitative responses from MammoWave raw-data frequency responses for an automated classification in WF and NF breasts, engaging the support vector machine (SVM) with radial basis function (RBF) kernel. The procedure is verified using clinical data collected in 61 breasts, each one having conventional exams by radiologists (which was used as the gold standard for our investigation). The contributions of the study are:

- The experimentation was completed on 61 breasts, allowing the exploration of lesions with different dimensions.
- The newly collected data appear differently in the hyperplane, motivating the authors to explore a radial basis function (RBF) kernel of SVM instead of a quadratic kernel, where SVM with an RBF kernel is found to be more efficient.
- The optimal method for using the frequency response signals was explored. The experiment shows that the 50 components obtained by applying a principal component analysis (PCA) from the real-parts of the $S_{21}$ parameters (engaging SVM with an RBF kernel) is the best possible combination to classify NF and WF signals.
- The prediction results have been analyzed by the team of researchers and radiologists through statistical measurements to understand the false positive and negative classifications, revealing that lesion size and breast density have an effect on microwave response, as well as ML predictions.

## 2. Methods

**Figure 1:**
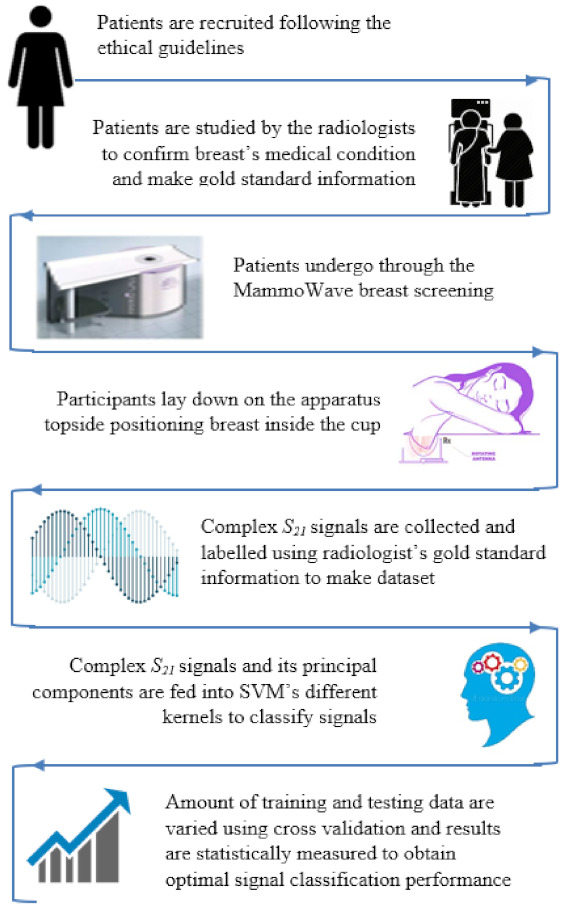
Proposed flow chart for the machine learning-based breast lesion detection.

A diagrammatic flow chart of the proposed work is shown in Figure 1. In more detail, each breast has its own correspondent output of the radiologist’s study review, which has been used as gold standard for the classification of the breasts in two categories: breasts with no radiological finding (NF), and breasts with radiological findings (WF), i.e., with lesions which may be benign or malignant. Gold standard labels of the breasts (NF or WF) have been employed to train and test the ML algorithms to identify microwave signals backscattered from the breasts automatically via the MammoWave.

## 3. Device Description

**Figure 2:**
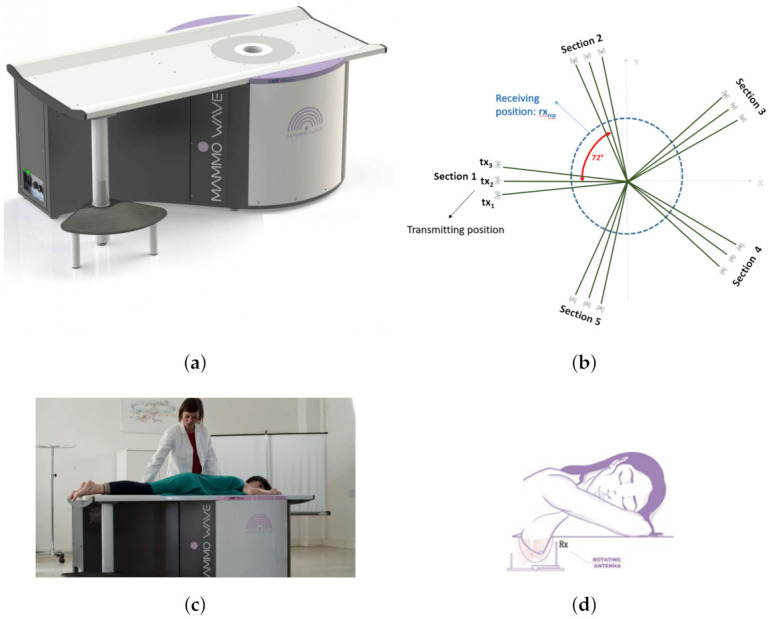
MammoWave device with patient’s position and transmitting-receiving antenna positions. (**a**) Novel MammoWave device. (**b**) Transmitting and receiving antenna rotation positions. (**c**) Patient’s posture over MammoWave. (**d**) Antenna rotation around the breast.

MammoWave (shown in Figure 2a) employs low power (1 mW) microwave signals in the 1–9 GHz frequency band. The device contains two antennas (Figure 2d) held in free space, which illuminates the breast using electromagnetic signals and measures the correspondent scattered electromagnetic fields from different angular positions around the azimuth. The two antennas are connected to a 2-port VNA (Cobalt C1209, Copper Mountain, Indianapolis, IN, USA). For each breast, measurements have been performed, recording the complex $S_{21}$, i.e., a parameter which is proportional to the electromagnetic field emerging from the transmitting antenna to the receiving one, after having interacted with the breast. The complex $S_{21}$ is recorded in a multi-bistatic fashion, i.e., for each transmitting position $tx_{m}$, the receiving antenna is moved to measure the received signal at the receiving points $rx_{n}$. In the current set-up, the receiving points are equally spaced at every 4.5${}^{\circ }$, leading to a total of $N_{RX}=80$ receiving points, Figure 2b. Concerning the transmitting positions, all experiments have been executed, employing $N_{TX}$ = 15 transmitting positions, displaced in 5 triplets, i.e., sections, centered at 0${}^{\circ }$, 72${}^{\circ }$, 144${}^{\circ }$, 216${}^{\circ }$, and 288${}^{\circ }$; in each triplet, the transmitting positions are displaced by 4.5${}^{\circ }$. For each transmitting and receiving position, the complex $S_{21}$ is collected from 1 to 9 GHz, with 5 MHz sampling (leading to NF=1601 frequency samples *f*). It follows, that for each breast, the raw data is represented by a matrix of complex $S_{21}$, having dimension $15\;\times \;80\;\times \;1601:S_{21}\left[N_{TX},N_{RX},NF\right]$. The exam is performed in less than 10 min per breast with the patient lying in a comfortable facing down position (shown in Figure 2c), with the breast (one at a time) positioned in a cup applying no compression. $S_{21}$ (i.e., raw data) may be then used to generate microwave images; however, in this paper, $S_{21}$ data only will be used.

## 4. Data Collection

The MammoWave feasibility clinical validation has been performed in Perugia Hospital and Foligno Hospital, Italy (Ethical Committee of Umbria, Italy, approval N. 6845/15/AV/DM of 14 October 2015, N. 10352/17/NCAV of 16 March 2017, N 13203/18/NCAV of 17 April 2018). All protocols and procedures were in accordance with both institutional and national ethical standards in research, and with the World Medical Association’s Declaration of Helsinki (1964) and its later amendments or analogous ethical standards. Prior to the trial, all participants have been requested to read and sign both the informative sheet and informed consent form.

This study comprises 61 breasts, 25 of which were found to be NF, and 36 were determined as WF, from 35 patients participating in the feasibility clinical trial. Microwave imaging was performed with patients who had already undergone a conventional radiologist’s examination review (used as a gold standard for our investigation). The average patient age was 52 years. Specifically, the radiologists reviewed conventional exams for each patient that agreed to participate in the study, classifying the breasts in to two groups: breasts with no radiological findings (NF) and breasts with radiological findings (WF), i.e., with lesions which may be benign or malignant. In this context, radiological study examination included: mammography, performed using a Selenia LORAD Mammography System (Hologic, Marlborough, MA), and/or echography, performed using the MyLab 70 xvg Ultrasound Scanner (Esaote, Genova, Italy), and/or magnetic resonance imaging, performed through a 3.0T MAGNETOM scanner (Siemens Healthcare, Erlangen, Germany). The lesion final assessment, performed using pathology within at least one year of clinical follow-up as reference standards was 22 benign lesions and 11 malignant lesions (while in three cases, the final assessment was not available). All lesion details are given in Table 1 (where possible, lesion details, dimensions, and lesion final assessment have been included).

## 5. MammoWave Signal Classification: Real-Parts of $S_{21}$ & RBF Kernel Approach

The raw frequency response includes the real and imaginary component, backscattered from the breast, i.e., $\lambda_{n}=\Sigma_{n=1}^{NF}{Real_{S}}_{21}\left(n\right)+j{Img_{S}}_{21}\left(n\right)$ where *n* is the number of frequencies, NF=1601, ${Real_{S}}_{21}$, and ${Img_{S}}_{21}$ represents the real and imaginary component, respectively. Initial studies performed by the authors on the MammoWave’s complex $S_{21}$ signal classification [21] indicates SVM with quadratic kernel ($SVM_{Q}$) is better able to categorise NF and WF signals over other tested conventional ML methods. Hence, this proposed work aims to further investigate the classification performance re-considering the real-parts of $S_{21}$ signals (in a form of $\Sigma_{n=1}^{NF=1601}{Real_{S}}_{21}\left(n\right)$) as feature values for NF-WF signal classification through SVM model. There are two ${Real_{S}}_{21}$ groups, NF and WF. A two-sample *t*-test has been performed to begin the experiment considering these groups. The *t*-test has been conducted to check whether the two types of ${Real_{S}}_{21}$ values are dependent and have equal variances. In other words, the outcome of the *t*-test signifies the suitability of ${Real_{S}}_{21}$ values for classifying NF and WF signals. The null hypothesis ($H_{0}$) here assumes that the two groups of ${Real_{S}}_{21}$ data samples are from populations with equal means. Therefore, the two types of ${Real_{S}}_{21}$ data samples can be employed for the classification task if the *t*-test rejects the $H_{0}$ and accept the alternative hypothesis ($H_{a}$). The alternative hypothesis ($H_{a}$) states that the ${Real_{S}}_{21}$ data comes from two different populations with unequal means. The desired significance level α=0.05 has been assumed for accepting and rejecting the null hypothesis, where the *p*-value has been compared for deciding the statistical significance. Furthermore, the confidence interval for the difference in population means of NF and WF’s ${Real_{S}}_{21}$ have been studied, where $C_{L}$ and $C_{U}$ demonstrate the lower and upper boundaries of the confidence interval. Table 2 shows the outcomes of the *t*-test, where p<α rejects the null hypothesis $H_{0}$ ($H_{0}=1$), accepts the alternative hypothesis $H_{a}$, and the true mean of the population belong between $-6.600\times 10^{-5}$ to $-4.600\times 10^{-5}$. Hence, the acceptance of the alternative hypothesis indicates that the ${Real_{S}}_{21}$ data comes from populations with unequal means and can be employed for the NF-WF signal classification task.

$Real_{S21}$ data of the NF and WF groups has been visualized in the 3D plane, which shows the spherical data, and might be classified better with the radial basis function (RBF) than the quadratic kernel of SVM. Thus, $SVM_{RBF}$ has been employed to classify NF and WF breast signals. The training and testing data have been divided using a Monte Carlo Cross Validation (MCCV) [29], where training and testing data have been initiated with 5% and 95%, respectively. The training data have been incremented by 5% in each simulation. The whole simulation has been repeated twenty-five times and average the performance metrics. $SVM_{RBF}$ computes the dissimilarity by measuring the squared Euclidean distance, which has been found to be more effective in the MammoWave breast classification task. Thus, the experiment has built on $SVM_{RBF}$ to improve the true positive prediction and reduce the false negative prediction.

**Figure 3:**
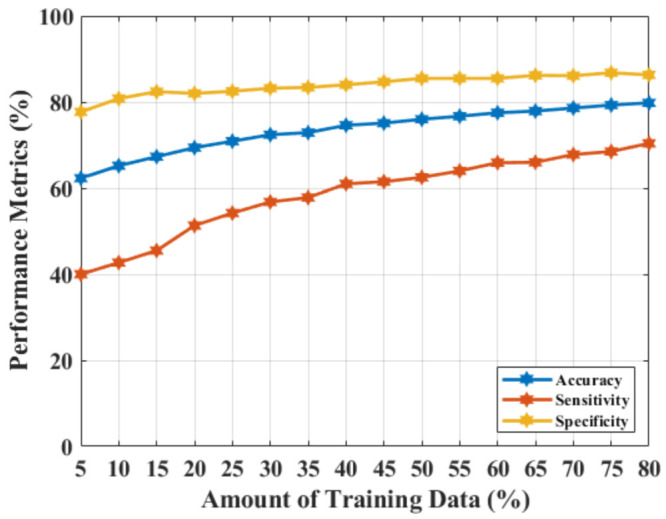
NF and WF signal prediction results (accuracy, sensitivity, and specificity) obtained using real-parts of the MammoWave’s frequency response and $SVM_{RBF}$, applying different amounts of training data.

Figure 3 shows the outcomes of the MammoWave signal classification for NF and WF detection using the real components of the complex signals. The accuracy, sensitivity, and specificity of 79.80%, 70.40%, and 86.30%, respectively were obtained, which indicate the real parts from the original feature dimension (real parts of Complex $S_{21}$) are not significant enough to be employed as features in this classification task, and may need feature extractions to improve the classification performance.

## 6. MammoWave Signal Classification Results: PCA on Real-Parts of $S_{21}$ & RBF Kernel Approach

**Figure 4:**
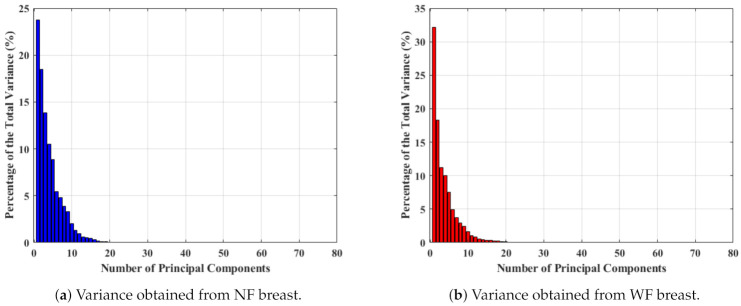
Percentage of variance obtained for 80 PCs measured from $Real_{S21}$.

Hence, one of the most popular feature extraction principal component analysis (PCA) technique was applied on $Real_{S_{21}}$ to transform more meaningful features for the classification task in a similar manner adopted to calculate the principal components (PCs) from the original complex signals of the MammoWave. Here, two vectors of variances (after PCA computation) have been selected from NF and WF breasts to study the magnitude of variance for selecting the number of PCs for the classification, as shown in Figure 4. Figure 4 shows the percentage of the total variance obtained from each PC for two different breast’s $S_{21}$, where first 80 PCs are found to be quantitatively significant. Hence, Figure 4a,b show the percentage of variance for the first 80 PCs, where the x−axis and y−axis represent the number of components and percentage of variance, respectively. Figure 4a displays the percentage of variance of an NF breast and Figure 4b describes the percentage of variance of a WF breast. As PCs show significant variance, up to 80 PCs ($\sigma_{1}$, $\sigma_{2}$,…, $\sigma_{80}$) have been selected for classification and varied, anticipating an improved performance.

The variance of PCs are close to each other in Figure 4. Two sample *t*-tests were constructed on the PCs to understand the capability to represent two signal groups and the data compactness, shown in Table 3. The probability has been found to be less than the significance level, p<α. Hence, the *t*-test accepts the alternative hypothesis $H_{a}$, and clearly demonstrates the presence of two different means for two different populations. Subsequently, the difference between the lower and upper boundary ($-1.770\times 10^{-4}$ and $-1.570\times 10^{-4}$) reduced, which implies an improved data compactness over the prior result.

**Figure 5:**
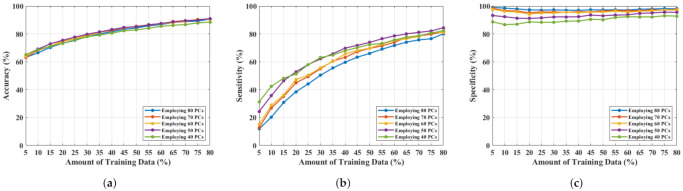
NF and WF breast signal classification results considering PCs measured from $Real_{S21}$ and employing SVM${}_{RBF}$, (**a**) accuracy, (**b**) sensitivity, (**c**) specificity.

An NF and WF signal classification has been performed by employing $SVM_{RBF}$ and varying the number of PCs from 80 to 40. The obtained results are shown in Figure 5. Figure 5a–c represents accuracy, sensitivity, and specificity, respectively, for applying a different number of PCs, where the *x*-axis represents the amount of training data used, and the *y*-axis describes the magnitude of the performance metric. The accuracy, sensitivity, and specificity have improved more than before, from 79.80% to 91%, 70.40% to 84.40%, and 86.30% to 97.20%. This is the optimal performance achieved, employing 50 PCs, and a further reduction of feature length (by 10 units of PCs) slopes down the classification metrics.

## 7. Discussion & Conclusions

The results demonstrate that a microwave breast imaging device (in this case MammoWave), when augmented by ML, could be employed to identify the presence of breast lesions with an accuracy of 91%, sensitivity > 84%, and specificity > 97%. Therefore, the augmentation of a non-ionizing and patient-comfort focused platform (MammoWave with ML) could be used to identify breast lesions in asymptomatic woman of any age and without any safety restrictions. This study comprises 61 breasts, of which 25 were NF and 36 were WF, from 35 patients participating for the feasibility clinical trial. Patients’ pre-menstrual information was not considered. False negative cases have been found in some cases, particularly in the presence of small sized lesions (<10 mm). This issue will be addressed in our future work, modifying the conventional $SVM_{RBF}$ kernel structure and performing advanced research on feature representation. Also, current WF breasts includes both benign and malignant lesions; three-classes breasts classification will be adopted (i.e., no finding, begin finding, and malignant finding) in future. ML experiment will be continued with ongoing clinical trial data [30] for enhancement in decision making process and helping in breast lesion identification for asymptomatic women of any age and without any safety restrictions.

## Figures and Tables

**Table 1:** Subject lists, details, and related radiologist’s review.

Age	Breast (L/R)	ACR Breast Density	Mammography BI-RADS	Echography BI-RADS	Radiologist’s Output Details: Sizes (mm) and Notes (If Available)	Pathology or 1-Year Clinical Follow-Up Output
48	L	D	3	-	Microcalcifications	Benign
65	L	C	4	-	Cluster of microcalcifications	Benign
40	L	B	2	2	Three masses: 15 mm, 21 mm, and 23 mm	Benign
	R	B	2	2	Microcalcifications	Not available
52	L	C	5	-	Microcalcifications	Malignant
47	L	D	2	2	Microcalcifications	Benign
55	R	C	2	2	1.6 mm microcalcifications	Benign
	L	C	2	2	3.8 mm microcalcifications	Benign
51	L	C	2	2	Presence of metallic marker	Benign
54	R	A	2	2	Microcalcifications	Benign
77	R	D	-	5	17 mm mass	Malignant
61	R	C	4	-	Multifocal lobular type suspected carcinoma (MRI BI-RADS 4)	Malignant
	L	C	2	-	Macrocalcification and Focal contrast enh. (MRI BI-RADS 3)	Not available
50	L	B	2	2	10 mm mass	Benign
67	L	C	4	-	Microcalcifications	Malignant
49	L	A	3	-	Microcalcifications	Benign
70	L	D	3	4	Mass	Malignant
42	L	C	2	3	7 mm mass, hypoechoic	Benign
67	L	B	3	-	Architectural distortion	Benign
56	R	B	4	4	31 mm mass, hypoechoic, irregular borders	Malignant
43	R	D	1	3	12 mm mass	Benign
51	L	C	3	-	Microcalcifications	Benign
59	L	B	-	4	11 mm areolar, suspicious of malignancy	Malignant
40	L	D	2	2	30 mm mass	Benign
35	R	C	2	3	7 mm, hypoechoic	Benign
37	L	A	2	3	25 mm mass	Benign
43	R	B	3	2	Microcalcifications	Malignant
54	R	B	2	2	18 mm mass	Benign
49	L	A	2	3	16 mm mass	Benign
56	L	D	4	4	27 mm mass	Malignant
63	L	A	3	4	6 mm mass	Malignant
55	R	C	4	4	23 mm mass	Malignant
	L	C	2	2	Multiple cysts	Benign
64	R	B	3	-	1.6 mm microcalcifications	Benign
37	R	-	-	3	15.4 mm mass	Benign
	L	-	-	2	Multiple cysts	Not available

**Table 2:** Two-sample *t*-test on real-parts of MammoWave’s $S_{21}$ data.

Null Hypothesis ($H_{0}$)	Probabilty (*p*)	Confidence Interval-Lower Boundary ($C_{L}$)	Confidence Interval-Upper Boundary ($C_{U}$)
1	$8.864\times 10^{-27}$	$-6.600\times 10^{-5}$	$-4.600\times 10^{-5}$

**Table 3:** Two-sample *t*-test on PCA features extracted from real-parts of MammoWave’s $S_{21}$ data.

Null Hypothesis ($H_{0}$)	Probabilty (*p*)	Confidence Interval-Lower Boundary ($C_{L}$)	Confidence Interval-Upper Boundary ($C_{U}$)
1	$8.219\times 10^{-230}$	$-1.770\times 10^{-4}$	$-1.570\times 10^{-4}$

## Data Availability

The datasets that support the findings of this study are not publicly available, but will be made available upon reasonable request, following ethics committee approval and a data transfer agreement to guarantee the General Data Protection Regulation. Please contact the authors, Dr. Soumya Prakash Rana (Email: ranas11@lsbu.ac.uk, soumyaprakash.rana@gmail.com), or Dr. Gianluigi Tiberi (Email: tiberig@lsbu.ac.uk, gianluigi@ubt-tech.com) to request access to the data.
